# Self-reported mask-related worrying reduces relative avoidance bias toward unmasked faces in individuals with low Covid19 anxiety syndrome

**DOI:** 10.1186/s41235-021-00344-8

**Published:** 2021-11-21

**Authors:** Anand Krishna, Johannes Rodrigues, Vanessa Mitschke, Andreas B. Eder

**Affiliations:** Lehrstuhl Für Psychologie II, Julius-Maximilians-Universität Würzburg, Röntgenring 10, 97070 Würzburg, Germany

**Keywords:** Approach-avoidance, Covid19, Masks, Anxiety

## Abstract

Facial masks have become and may remain ubiquitous. Though important for preventing infection, they may also serve as a reminder of the risks of disease. Thus, they may either act as cues for threat, priming avoidance-related behavior, or as cues for a safe interaction, priming social approach. To distinguish between these possibilities, we assessed implicit and explicit evaluations of masked individuals as well as avoidance bias toward relatively unsafe interactions with unmasked individuals in an approach-avoidance task in an online study. We further assessed Covid19 anxiety and specific attitudes toward mask-wearing, including mask effectiveness and desirability, hindrance of communication from masks, aesthetic appeal of masks, and mask-related worrying. Across one sample of younger (18–35 years, *N* = 147) and one of older adults (60+ years, *N* = 150), we found neither an average approach nor avoidance bias toward mask-wearing compared to unmasked individuals in the indirect behavior measurement task. However, across the combined sample, self-reported mask-related worrying correlated with reduced avoidance tendencies toward unmasked individuals when Covid19 anxiety was low, but not when it was high. This relationship was specific to avoidance tendencies and was not observed in respect to explicit or implicit preference for mask-wearing individuals. We conclude that unsafe interaction styles may be reduced by targeting mask-related worrying with public interventions, in particular for populations that otherwise have low generalized Covid19 anxiety.

**Supplementary Information:**

The online version contains supplementary material available at 10.1186/s41235-021-00344-8.

## Introduction

During the Covid19 pandemic, the use of protective facemasks to limit infection has become commonplace (Haischer et al., 2020). In many countries, wearing a mask is mandatory in certain public spaces (Adjodah et al., 2021). Although masks are effective in reducing person-to-person transmission (Adjodah et al., 2021; Eikenberry et al., 2020), many individuals have a negative view of mask-wearing (Mallinas et al., 2021). Such individuals are less likely to report following mask-wearing recommendations and mandates (Rieger, 2020) and may be more inclined toward other risky behaviors, such as preferentially seeking contact with non-mask wearers. However, measuring such behavior is challenging. Research focusing on mask-wearing behavior in general has mostly relied on self-report measures (Asri et al., 2021; Betsch et al., 2020; Xu & Cheng, 2021) or approached the question from epidemiological, non-psychological perspectives (Adjodah et al., 2021).

Approach-avoidance motivation research offers an avenue into measuring potential interaction styles toward mask-wearing individuals. Approach motivation deals with behavior directed at achieving positive outcomes, whereas avoidance motivation deals with behavior that seeks to evade negative outcomes (Elliot et al., 2013). Action tendencies associated with approach and avoidance motivations can be measured with simplified computerized approach-avoidance tasks (AATs) that measure the speed of pull–push movements (e.g., Solarz, 1960), button presses (e.g., Aubé et al., 2019), and whole-body movements (e.g., Eder et al., 2021) in response to motivationally relevant stimuli (for meta-analyses see Beatty et al., 2016; Laham et al., 2015; Phaf et al., 2014). Studies demonstrate that the predominant action tendency measured with these tasks predicts a range of complex human behaviors underlying self-control (e.g., Fishbach & Shah, 2006), consumer decisions (e.g., Bradley et al., 2008), social approach (e.g., Roelofs et al., 2009), and psychopathology (e.g., Lange et al., 2008; Najmi et al., 2010).

Keeping distance from individuals wearing no protective face mask is particularly important during Covid19 to reduce the spread of the coronavirus. Many countries have therefore implemented social distancing rules. Given that masks effectively reduce the risks of contagion (Adjodah et al., 2021; Eikenberry et al., 2020), a mask-wearing person could signal safety and compliance with public health regulations, facilitating social approach. However, the perception of a face mask could also prime unpleasant cognitions of infection, discomfort, or difficulty in communicating, facilitating social avoidance. Accordingly, dominant tendencies for avoidance or approach are *both* theoretically plausible for social interactions with individuals wearing a face mask. In addition, to the degree that an individual’s beliefs and attitudes about mask-wearing are reflective of the accessibility of such cognitions (Fazio et al., 1986), these attitudes should be related to the relative dominance of approach-avoidance predispositions.

Research has shown that risk aversion and anxiety reduction correlate with self-reported distancing and mask use (Nakayachi et al., 2020; Xu & Cheng, 2021), with older individuals reporting more mask-wearing habits when more concerned with risk to the self and younger individuals being more influenced by concerns with others (Asri et al., 2021). However, concerns with aesthetic aspects of wearing masks and aversion to wearing them also predicted the self-reported likelihood of mask wearing (Rieger, 2020). While none of these studies specifically examined approach-avoidance tendencies toward mask-wearing individuals, they are consistent with an impact of beliefs and anxiety on health-related behavior. Thus, positive attitudes toward aspects of mask-wearing may promote approach responses toward individuals wearing masks, while negative attitudes might conversely increase avoidance responses. A predisposition to avoid unmasked individuals more than unmasked individuals would be desirable from a health policy standpoint, as this would imply a decreased likelihood of interacting with unmasked individuals, where the likelihood of contagion is higher. Thus, the current research investigates predictors of such tendencies.

We know of only one unpublished study that has investigated approach-avoidance responses to mask-wearing faces (Ingram et al., 2021). The results showed an approach bias toward mask-wearing faces in a large sample, but they do not provide any insight on what attitudes might relate to this bias. Thus, we additionally measured different aspects of participants’ positive and negative attitudes toward wearing masks using implicit and explicit measurement tasks as well as their generalized anxiety about Covid19. The attitude scores were then related to indices of behavioral predispositions to approach versus avoid (un)masked individuals as measured with the AAT. We sampled from both young adult and older adult populations, as previous research indicates that older adults suffered from less anxiety during the pandemic (Solomou & Constantinidou, 2020), but at the same time were at higher risk (Zhou et al., 2020) and may be targeted more by public safety announcements (Daoust, 2020; see also Utych & Fowler, 2020). Thus, there may be differences in their relative anxiety levels with regard to Covid19, which may in turn affect approach-avoidance tendencies. In order to maximize the chance of finding such differences, we limited data collection to the youngest and oldest age categories applied in previous studies (Solomou & Constantinidou, 2020).

## Method

We conducted two exploratory studies of similar design. Although we had no specific directional predictions, we preregistered the materials, methodology, and general analysis plan with data exclusion rules and exploratory analyses for each study (Study 1a: https://osf.io/xk5wz, Study 1b: https://osf.io/grh3w).

### Sample, design, and procedure

Both studies collected data from English-speaking participants on the Prolific Academic website, an international recruitment platform for paid participants, between January 22 and February 02, 2021. Study 1a was limited to participants under 35 (initial sample *N* = 171, 15 incomplete datasets, three high error-rates in the AAT, three excessive durations, three missed attention checks; final *N* = 147; 60 female, 86 male, 1 diverse; age: *M* = 24.0, SD = 4.5, Min = 18, Max = 35), whereas Study 1b was limited to participants over 60 (initial sample *N* = 175, one below 60 years of age, 17 incomplete datasets, one high error-rate in the AAT, two excessive durations, four missed attention checks; final *N* = 150; 72 female, 78 male; age: *M* = 65.3, SD = 4.7, Min = 60, Max = 83). Both samples were majority European, although it should be noted that the older sample in particular was heavily skewed toward the United Kingdom (young sample: 118 from EU countries, 18 from UK, 11 from other countries; older sample: 6 from EU countries, 132 from UK, 13 from other countries). See Additional file 1 for more detailed distribution data.

The studies followed a 2-cell within-subjects design with the factor target type (unmasked vs. mask-wearing). After providing informed consent and demographic data, participants completed measures of explicit and implicit evaluations of unmasked and mask-wearing persons (order counterbalanced). Next, participants performed an AAT. Finally, they completed the explicit and implicit evaluation measures once again, followed by several explicit items measuring various attitude components toward individuals who wear masks.

### Material

The mask-wearing/unmasked persons were faces of 6 white men and 6 white women selected from the Chicago Face Database (Ma et al., 2015). An additional picture set of the faces was created with adding a standard one-use blue mask covering the nose and mouth. Each person was thus shown wearing a mask and without a mask.

### Evaluative measures

For the explicit preference measure, participants were asked whether they would prefer to spend time with the mask-wearing or unmasked version of the same person for each target person (order randomized, dichotomous choice).

For the implicit evaluation measure, participants completed a Brief Implicit Association Test (BIAT; Nosek et al., 2014) with the item categories unmasked faces and mask-wearing faces and the valence types positive and negative. In each trial, participants saw a target word or face after 150 ms. If the target belonged to the *focal* face category (e.g., mask-wearing face) or focal valence category (e.g., positive word), participants had to press the focal key, otherwise the non-focal key as fast as possible. If they responded incorrectly, a red X was displayed above the word or face and the trial continued until they responded correctly. Participants completed five BIAT blocks in which focal categories varied, each of which started with four trials containing only positive and negative words followed by 16 trials in which words alternated with pictures showing mask-wearing and unmasked persons in random order.

The response keys were the E and I keys on the keyboard. Key assignment (focal vs. non-focal) was counterbalanced across participants, with the focal key always being associated with positive valence. In the first practice block (12 trials), the category targets were words denoting either mammals or birds. In the following four BIAT blocks, the targets were the unmasked/masked faces and positive/negative words. These blocks alternated between *unmasked-positive/masked-negative* and *unmasked-negative/masked-positive* response key pairings by varying the response key for the unmasked/masked categories. The starting block mapping was counterbalanced across participants.

#### AAT

In the AAT, participants responded with approach/avoidance-related movements as quickly and as accurately as possible to a face shown at the center of the screen. In each trial, participants saw a black-and-white grid background showing a corridor. A fixation cross appeared at the center of the screen that was replaced by a face after 500 ms. Participants responded to the appearance of the face by pressing the up and down arrow keys on the computer keyboard as quickly as possible. Responses triggered zooming effects indicative of approach or avoidance movements (up: movement toward face, down: movement away from face) for 18 frames, remaining at the final distance for 500 ms. Error feedback appeared for 4000 ms if the participant pressed the wrong key or did not react within 2000 ms. The next trial began after 150 ms.

Participants completed two blocks of this task of 60 trials (30 unmasked, 30 mask-wearing) each. For one block, task instructions were to approach unmasked and avoid mask-wearing faces; for the other block, this mapping was reversed (order counterbalanced).

### Mask attitude items

Fifteen self-generated items measured opinions about mask-wearing on 7-point Likert scales from 1 (“disagree completely”) to 7 (“agree completely”), presented in randomized order (see Additional file 1 for item wording). Two attention check questions were randomly interspersed with the mask attitude items.

### COVID-19 Anxiety Syndrome Scale

Next, participants were administered the COVID-19 Anxiety Syndrome Scale (C-19 ASS, Nikčević & Spada, 2020), comprising 9 items measuring frequency of anxiety responses in regard to infection with Covid-19 on a 5-point scale (from 0 “Not at all” to 4 “Nearly every day”). In Study 1b, participants additionally responded to the item: “How high would you estimate your personal risk of becoming infected with Covid19?” (Likert response from 1—“negligible risk” to 5—“moderate risk” to 9—“very high risk”). This item was not analyzed.

## Results

Raw data and analysis scripts are available under https://osf.io/q8sed/. Data preparation in accordance with our preregistration is detailed in Additional file 1. Descriptive statistics for all test-relevant variables are shown in Table 1.

**Table 1:** Descriptive data

	Young sample		Older sample		Combined	
	*M*	SD	*M*	SD	*M*	SD
Explicit mask preference (pre AAT)	2.61	4.30	0.52	4.79	1.55	4.67
Explicit mask preference (post AAT)	2.71	4.37	0.45	5.11	1.57	4.88
Explicit mask preference (averaged)	2.66	4.23	0.49	4.82	1.56	4.66
BIAT d score (pre AAT)	0.18	0.41	0.10	0.41	0.14	0.41
BIAT d score (post AAT)	0.11	0.38	0.06	0.37	0.09	0.38
BIAT d score (averaged)	0.15	0.33	0.08	0.32	0.11	0.32
Avoidance bias for unmasked faces	2 ms	51	4 ms	67	3 ms	59
Covid 19 Anxiety Syndrome Scale (C-19 ASS) scores	2.00	0.88	1.83	0.89	1.91	0.89
Mask effectiveness	5.94	1.08	6.04	1.14	5.99	1.11
Aesthetic appeal	3.62	1.42	2.91	1.43	3.26	1.46
Mask-related worrying	2.74	1.17	3.20	1.29	2.97	1.25
Communication difficulties	3.52	1.45	4.67	1.41	4.10	1.54

Explicit mask preference scores ranged from − 6 to 6. Covid 19 Anxiety Syndrome Scale scores ranged from 0 to 4. Mask effectiveness, aesthetic appeal, mask-related worrying, and communication difficulties scores ranged from 1 to 7

### Preregistered analyses

We preregistered specific analyses for avoidance bias scores to test for any directed tendency. In addition, we preregistered analyses testing the explicit and implicit evaluation measures for equivalence before and after the AAT in order to rule out any effects of the procedure on attitudes.

#### AAT

Avoidance bias scores were subjected to two-tailed one-sample *t *tests against 0. For both studies, the results indicated that there was no difference from zero (young sample: *t*(146) = 0.56, *p* = 0.574, *d* = 0.004, BF_01_ = 9.32; older sample: *t*(149) = 0.79, *p* = 0.433, *d* = 0.064, BF_01_ = 8.12).

#### Evaluation measures

For the explicit preference measure, a paired-samples Bayesian t test indicated moderate to strong support for a null difference pre- and post-AAT (young sample: BF_01_ = 8.61, MD[*δ*] = − 0.056, CI[δ]_95%_ = [− 0.219, 0.105]; older sample: BF_01_ = 10.33, MD[*δ*] = 0.029, CI[*δ*]_95%_ = [− 0.187, 0.130]). For the d-scores, two participants were eliminated from analysis in Study 1a and none in Study 1b due to having at least two BIAT blocks with high percentages of fast responses as preregistered. There was an inconclusive result for the young sample (BF_01_ = 1.54, MD[*δ*] = 0.163, CI[*δ*]_95%_ = [0.001, 0.325]), but the predicted null difference was supported moderately for the older sample (BF_01_ = 7.27, MD[*δ*] = 0.073, CI[*δ*]_95%_ = [− 0.085, 0.228]). Implicit and explicit evaluations appeared mostly robust to any changes due to the AAT.

### Exploratory analyses

Bayesian analyses of avoidance bias and BIAT d-scores showed no evidence for differences between the young and older samples (both BF_01_ ≥ 1.55), although the explicit preference for mask-wearing individuals was greater in the young sample than in the older sample, *t*(295) = 5.70, *p* < 0.001, *d* = 0.479, BF_10_ = 363.52. In order to maximize power with view to our main research question (predicting avoidance bias), all exploratory analyses were conducted on the combined sample (total *N* = 297; *N* = 295 for d-score analyses). A one sample t test of avoidance bias on the combined sample against zero indicated no difference to zero, *t*(296) = 0.97, *p* = 0.334, *d* = 0.056, BF_01_ = 9.67. However, participants preferred mask-wearing faces both in the explicit ratings, *t*(296) = 5.78, *p* < 0.001, *d* = 0.335, BF_10_ = 414,457.16, and BIAT d-scores, *t*(294) = 6.11, *p* < 0.001, *d* = 0.356, BF_10_ = 2.36e+6.

We extracted mask attitude components from our self-generated items using an exploratory factor analysis (see Additional file 1 for details). Independent samples t tests for Covid19 anxiety between younger and older adults showed a nonsignificant tendency for lower anxiety on the C-19 ASS scores among older adults, *t*(295) = 1.73, *p* = 0.085, *d* = 0.201, BF_01_ = 1.88, but significantly higher mask-related worrying among older adults, *t*(295) = 3.19, *p* = 0.002, *d* = 0.370, BF_10_ = 15.52. This suggests that momentary anxious responses are dependent on age.

Effects of mask attitude components on explicit and implicit attitudes and AAT bias scores, including C-19 ASS scores as an exploratory predictor and moderator, were analyzed using a stepwise general linear model (GLM) approach. The results are shown in Tables 2, 3 and 4.

**Table 2:** Stepwise general linear model with explicit evaluation of (masked) faces as outcome

Predictor	Model 1 *R*^2=^.341 AIC = 1644			Model 2 *R*^2=^.351 AIC = 1642			Model 3 *R*^2=^.355 AIC = 1648		
	*B*	SE	*p*	*B*	SE	*p*	*B*	SE	*p*
Intercept	− 3.50	1.84	.059	− 3.39	1.83	.065	− 1.24	3.93	.752
Mask effectiveness	1.08	.23	< .001	.89	.24	< .001	.73	.43	.088
Aesthetic appeal	.77	.17	< .001	.76	.16	< .001	.85	.40	.035
Mask-related worrying	− .50	.20	.015	− .59	.21	.005	− .51	.50	.305
Communication difficulties	− .59	.16	< .001	− .55	.16	< .001	− .95	.39	.015
C-19 ASS				.58	.27	.033	− .45	1.97	.819
Mask effectiveness * C-19 ASS							.08	.23	.718
Aesthetic appeal * C-19 ASS							− .06	.18	.746
Mask-related worrying * C-19 ASS							− .04	.23	.854
Communication difficulties * C-19 ASS							.20	.17	.237

Explicit mask preference scores ranged from − 6 to 6. Covid 19 Anxiety Syndrome Scale scores ranged from 0 to 4. Mask effectiveness, aesthetic appeal, mask-related worrying, and communication difficulties scores ranged from 1 to 7

**Table 3:** Stepwise general linear model with implicit evaluation of (masked) faces (BIAT d-scores) as outcome

Predictor	Model 1 *R*^2=^.073 AIC = 159			Model 2 *R*^2=^.076 AIC = 160			Model 3 *R*^2=^.085 AIC = 165		
	*B*	SE	*p*	*B*	SE	*p*	*B*	SE	*p*
Intercept	.10	.15	.498	.11	.15	.475	.44	.33	.173
Mask effectiveness	.04	.02	.024	.04	.02	.083	.01	.04	.681
Aesthetic appeal	− .02	.01	.262	− .02	.01	.257	− .02	.03	.631
Mask-related worrying	− .01	.02	.385	− .02	.02	.299	− .07	.04	.075
Communication difficulties	− .04	.01	.006	− .04	.01	.009	− .05	.03	.156
C-19 ASS				.02	.02	.342	− .15	.17	.361
Mask effectiveness * C-19 ASS							.01	.02	.533
Aesthetic appeal * C-19 ASS							> − .00	.01	.949
Mask-related worrying * C-19 ASS							.03	.02	.139
Communication difficulties * C-19 ASS							.01	.01	.729

Covid 19 Anxiety Syndrome Scale scores ranged from 0 to 4. Mask effectiveness, aesthetic appeal, mask-related worrying, and communication difficulties scores ranged from 1 to 7

**Table 4:** Stepwise general linear model with avoidance bias scores as outcome

Predictor	Model 1 *R*^2=^.032 AIC = 3270			Model 2 *R*^2=^.035 AIC = 3271			Model 3 *R*^2=^.090 AIC = 3261		
	*B*	SE	*p*	*B*	SE	*p*	*B*	SE	*p*
Intercept	− 46.45	28.45	.104	− 45.71	28.46	.109	7.67	59.39	.897
Mask effectiveness	7.72	3.50	.028	6.37	3.76	.091	9.38	6.44	.147
Aesthetic appeal	2.10	2.54	.755	2.08	2.54	.613	4.69	6.09	.019
Mask-related worrying	− .97	3.12	.409	− 1.62	3.19	.415	− 17.76	7.53	.443
Communication difficulties	− .11	2.47	.964	.21	2.49	.932	− 7.01	5.83	.230
C-19 ASS				4.19	4.24	.324	− 7.45	29.85	.803
Mask effectiveness * C-19 ASS							− 3.20	3.46	.356
Aesthetic appeal * C-19 ASS							− 1.92	2.68	.030
Mask-related worrying * C-19 ASS							7.49	3.44	.476
Communication difficulties * C-19 ASS							3.60	2.62	.170

Covid 19 Anxiety Syndrome Scale scores ranged from 0 to 4. Mask effectiveness, aesthetic appeal, mask-related worrying, and communication difficulties scores ranged from 1 to 7

#### GLM analyses of explicit face evaluations

The model including the main effects of attitude components and C-19 ASS achieved the lowest AIC score (indicating the best model fit). Although there was no significant difference from the model excluding the C-19 ASS effect (*p* = 0.368), including the C-19 ASS score increased the proportion of explained variance (Δ*R*^2^ = 0.010, *F*(1,291) = 4.57, *p* = 0.033). In general, higher positive mask attitude components (mask effectiveness, aesthetic appeal) and general Covid anxiety were positively and higher negative mask attitude components were negatively linking to preference for spending time with masked individuals.

#### GLM analyses of implicit face evaluations

For implicit evaluations (BIAT d-scores), the model including only the attitude component main effects achieved the lowest AIC score, although this did not differ significantly from the model including the C-19 ASS effect (*p* = 0.607). As the latter provided no increase in explained variance (Δ*R*^2^ = 0.003, *F*(1,289) = 0.91, *p* = 0.342), only the main effects of attitude components are discussed. Implicit preference for mask-wearing faces increased with mask effectiveness scores and weakened with communication difficulties scores.

#### GLM analyses of avoidance bias scores

**Fig. 1:**
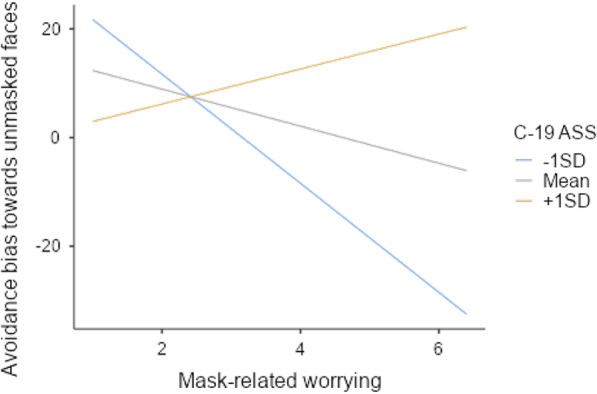
Regression model estimates of avoidance bias based on mask-related worrying at varying levels of COVID-19 anxiety (C-19 ASS scores)

The model including the interaction terms achieved the lowest AIC, differing significantly from the other two models (*p* ≤ 0.011). Mask-related worrying and its interaction with C-19 ASS scores were significant. High endorsement of mask-related worrying was linked to a diminishing avoidance bias for individuals with low C-19 ASS scores, whereas for individuals high in C-19 ASS, this did not hold (see Fig. 1). Individuals who endorsed mask effectiveness items also tended toward showing an avoidance bias in the attitude components main effects model; however, this finding was not robust to the addition of additional predictors.

## Discussion

We assessed attitude components toward wearing protective face masks and Covid19 anxiety syndrome (Nikčević & Spada, 2020), as well as implicit and explicit preferences for mask-wearing individuals and tendencies to avoid unmasked individuals using an AAT. Participants preferred mask-wearing individuals both in implicit and explicit measures, but this did not translate to a general avoidance of unmasked individuals. Favorable attitudes about wearing protective face masks and high levels of Covid19 anxiety predicted explicit preferences for spending time with mask-wearing individuals. Specific attitudes about the effectiveness and desirability of wearing masks and in respect to communication difficulty predicted implicit preferences for people wearing masks positively and negatively, respectively. However, there was only weak evidence for a positive impact of mask effectiveness and none for any effect of communication difficulties on avoidance bias toward unmasked individuals. Instead, fears and worries related to seeing another person wearing a protective mask reduced social avoidance tendencies toward unmasked individuals only when Covid19 anxiety was low, but not when Covid19 anxiety was high. Finally, no robust difference in generalized Covid19 anxiety was found between younger and older adults, but older adults reported greater mask-related worrying.

These results show the importance of understanding how specific attitude aspects toward mask-wearing may influence different safety-relevant behaviors. While previous research has demonstrated that self-reported mask-wearing or -buying intentions depend on individuals’ attitudes toward masks (Rieger, 2020; Shah et al., 2020), our results indicate that social interactions with mask-wearing individuals may also be affected on a behavioral level. Practical aspects such as effectiveness and desirability of mask-wearing and expected communication difficulties affected automatic evaluations of mask-wearing individuals, but most importantly, the automatic tendency to avoid unmasked individuals relative to mask-wearing individuals was affected by mask-related worrying, including nervousness, fears of infection, and anticipated discomfort. This suggests that public interventions to reduce such worries caused by masks may be a helpful tool to reduce unsafe interactions.

Our finding that mask-related worrying only reduces avoidance bias toward unmasked individuals in participants with low Covid19 anxiety specifies this further. It seems that mask-related worrying only determines avoidance bias in the absence of elevated fear of infection. Thus, strong infection anxiety may supersede any negative effect of mask-related worries on unsafe social interaction tendencies. However, as vaccination rates increase, fear of infection is likely to decrease, even though vaccines may offer reduced protection against some virus variants (Madhi et al., 2021; Shinde et al., 2021). Thus, public messaging that aims to increase the association of safety rather than worry with masks is likely to pay dividends in the foreseeable future.

Some limitations of our study must be noted. First, our design does not allow us to make causal statements. Although it is theoretically plausible that attitude components determine approach bias, behavior may also influence attitudes (Bem, 1972). Second, our relative avoidance bias measure does not differentiate between a tendency to avoid unmasked individuals and a tendency to approach mask-wearing individuals. However, the latter tendency would also not be optimal, because wearing a mask does not compensate for the increased risk of infection in close distance (Kwon et al., 2021; Li et al., 2021). Third, spontaneous approach-avoidance tendencies toward individuals wearing a face mask were assessed using a computerized reaction time task (AAT). While the construct validity of this task in social-personality and clinical domains has been demonstrated by many research findings (e.g., Neumann et al., 2004; Rinck & Becker, 2007), it is not clear whether the effects obtained with a manual task could be generalized to other social behaviors occurring outside of the laboratory, such as those involving the whole body (Eder et al., 2021; Stins et al., 2011). Future research could use other AAT variants to investigate this issue, although there is some evidence of AAT effects generalizing to actual social interactions (Taylor & Amir, 2012). Fourth, our sample was recruited online, which limits its representativeness. Our findings are skewed toward wealthier countries with better Internet access and should not be uncritically applied to populations with less Internet access or in countries where such access is less common, as both information about Covid19 and public messaging would likely differ significantly for these populations. In addition, the European focus of our participants may make generalization to US populations problematic. In a similar vein, our use of only Caucasian faces in our study may limit its applicability to non-White populations. However, as we sampled majority-White countries, it is theoretically somewhat plausible that our results would likely generalize to interactions with the majority racial groups in other countries. Finally, we recruited no participants of ages between 35 and 60 years. While we thus cannot definitively state that our findings will generalize to this age group, previous research does indicate a linear downwards trend for anxiety and depression during the pandemic across it (Solomou & Constantinidou, 2020). Thus, it seems unlikely that the middle-aged group would show major departures from our findings.

In conclusion, we show that seeing others wearing masks can cause specific worries and anxieties in individuals that in turn may reduce their relative avoidance of relatively risky interactions with unmasked individuals. Thus, interventions aimed at reducing such risky contacts should focus on these worries, particularly in populations that otherwise do not show generalized Covid19 anxiety.

### Significance statement

Face masks can protect from infection, but some people may prefer to interact with unmasked individuals, leading to increased risks. Measurements of spontaneous action tendencies toward mask-wearing vs. unmasked individuals, attitudes toward people wearing masks, and associated risks of disease transmission should provide insight as to whether people will avoid relatively unsafe interactions with unmasked individuals. We measured behavioral approach-avoidance tendencies using a computerized approach-avoidance task, as well as peoples’ spontaneous relative evaluations of mask-wearing versus unmasked faces and their explicit preference with whom they would rather spend time. We found no relative avoidance tendency toward unmasked individuals in the approach-avoidance task, although people did report preferring spending time with mask-wearing individuals and spontaneously responded more positively toward them. Participants’ general beliefs about mask effectiveness, aesthetic appeal of masks, mask-related worrying, and communication difficulties associated with masks as well as their general anxiety about Covid were all associated with their preference to spend time with mask-wearing individuals. However, their spontaneous evaluations were linked only to their beliefs about mask effectiveness and communication difficulties. On the other hand, avoidance tendencies toward unmasked individuals were linked only to mask-related worrying (e.g., nervousness from seeing masks), specifically for participants who had low general anxiety about Covid (and would therefore be less likely to balance mask-related worries with relatively less fear of infection due to masks). We conclude interventions targeting mask-related worrying will be most effective in keeping people from risky interactions with unmasked individuals, especially in groups where general Covid19 anxiety is low.

## Supplementary Information

**Additional file 1.** Expanded sample demographics, preregistered data preparation, and factor analysis.

## Data Availability

All data and materials underlying this research are available on the OSF. Materials can be found in the ‘Files’ section of the preregistrations. The preregistrations for the experiments may be found at https://osf.io/xk5wz (Study 1a) and https://osf.io/grh3w (Study 1b). Raw data and analysis scripts are available at https://osf.io/ec2h4/ (Study 1a) and https://osf.io/q8sed/ (Study 1b, including combined analyses).
