# Supplementary material for: Self-reported mask-related worrying reduces relative avoidance bias toward unmasked faces in individuals with low Covid19 anxiety syndrome
Source: Cogn Res Princ Implic. 2021 Nov 21;6:75. doi: 10.1186/s41235-021-00344-8 (PMC8606225; doi:10.1186/s41235-021-00344-8)
Supplement: Supplementary file 1 — Additional file 1. Expanded sample demographics, preregistered data preparation, and factor analysis. [file 41235_2021_344_MOESM1_ESM.docx]

# Supplementary Materials

## Supplementary Demographic Data

Supplementary Table 1 shows the distribution of participants across countries of residence per sample.

Supplementary Table 1. Number of participants by sample and country of residence.

| Country | *n (%)*  Younger sample | *n (%)*  Older sample |
| --- | --- | --- |
| Australia | - | 2 (1.3%) |
| Belgium | 2 (1.4%) | - |
| Canada | 2 (1.4%) | - |
| Chile | 2 (1.4%) | - |
| Czech Republic | 2 (1.4%) | - |
| Estonia | 2 (1.4%) | - |
| Finland | 1 (0.7%) | - |
| France | 2 (1.4%) | - |
| Germany | 2 (1.4%) | - |
| Greece | 6 (4.1%) | 1 (0.7%) |
| Hungary | 6 (4.1%) | - |
| Ireland | 2 (1.4%) | - |
| Israel | - | 1 (0.7%) |
| Italy | 25 (17.0%) | 2 (1.3%) |
| Malaysia | 1 (0.7%) | - |
| Mexico | 3 (2.0%) | - |
| Netherlands | 1 (0.7%) | - |
| Poland | 31 (21.1%) | - |
| Portugal | 31 (21.1%) | - |
| South Africa | 2 (1.4%) | 2 (1.3%) |
| Spain | 5 (3.4%) | 3 (2.0%) |
| Sweden | - | 1 (0.7%) |
| United Kingdom | 18 (12.2%) | 132 (87.4%) |
| USA | 1 (0.7%) | 7 (4.6%) |

## Preregistered Data Preparation

Explicit preference responses for mask-wearing faces were summed and centered around indifference, yielding a range from -6 to 6.

The first 12 trials of each AAT block were eliminated. After removal of reaction time outliers with individual Tukey criterions, latencies of correct reactions were averaged for each mapping and face type condition. For each participant, the average RT for approach-masked and avoid-unmasked was subtracted from the average RT for approach-unmasked and avoid-masked to calculate a relative avoidance bias towards unmasked individuals. Note that our preregistration named this index a relative avoidance bias towards masked individuals; the alternative label was chosen in the manuscript for ease of interpretation.

For the BIAT, the practice block and first four trials from all other blocks were eliminated. Reaction times were winsorized (lower bound 400ms, upper bound 2000ms) and d parameters were calculated separately for the block combinations two/three and four/five, then averaged to a final d parameter, coded such that positive values indicated greater implicit preference for mask-wearing faces (Nosek et al., 2014).

## Exploratory Factor Analysis

To investigate relevant attitude aspects, we first subjected our self-generated items to an exploratory factor analysis (EFA) using oblimin rotation and the minimum residuals extraction method. The number of extracted factors was based on parallel analysis. The initial EFA produced a four-factor solution in which two items cross-loaded on two factors (*r* > .3). In order to achieve simple structure and ensure interpretability, we removed these items and reran the EFA with the same parameters (Hair et al., 2018). This produced a four-factor solution in which one item cross-loaded on two factors. We removed this item and reran the EFA. This produced a four-factor solution with no cross-loadings. The factor loadings are shown in Supplementary Table 2.

Supplementary Table 2. Factor loadings after exploratory factor analysis.

| Item | Factor 1  Mask effectiveness | Factor 2  Aesthetic appeal | Factor 3  Mask-related worrying | Factor 4  Communication difficulties |
| --- | --- | --- | --- | --- |
| I am grateful to others who wear masks. | .930 | -.062 | .052 | .012 |
| Wearing masks is pointless, so I don’t like seeing others wearing them. | -.794 | .006 | .010 | .120 |
| It is admirable to wear masks. | .655 | .155 | -.067 | .221 |
| It annoys me when I see people wearing masks. | -.640 | .015 | .125 | .167 |
| Seeing people wearing masks makes me feel safe. | .613 | .162 | -.065 | .034 |
| People wearing masks is aesthetically pleasing to me. | -.005 | .761 | -.007 | .032 |
| Wearing masks makes people look attractive. | .015 | .758 | .035 | -.061 |
| Seeing people wear masks makes me feel nervous. | -.111 | .023 | .718 | .015 |
| Seeing people wear masks makes me fear the risk of infection. | .164 | .032 | .623 | -.021 |
| I am reminded of the discomfort of wearing a mask when I see others wearing them. | -.155 | -.238 | .322 | .157 |
| I have difficulty understanding people who are wearing masks. | -.068 | .006 | .010 | .697 |
| When people wear masks, it is more difficult to communicate with them. | .027 | -.293 | .111 | .480 |

*Note.* Oblimin-rotated exploratory factor analysis results after eliminating items “I like seeing people wear masks” and “Seeing people wearing masks reminds me of the difficulties the pandemic brings” in the first iteration and “Seeing people wear masks makes me feel sad” in the second iteration. Shaded cells indicate items assigned to a factor.

# References

Hair, J. F., Black, W. C., Babin, B. J., & Anderson, R. E. (2018). *Multivariate Data Analysis* (8th ed.). Cengage Learning EMEA.
